# Neurocardiac signatures of acute mental stress: a sex-comparative study

**DOI:** 10.3389/fnins.2025.1633295

**Published:** 2025-08-13

**Authors:** Selina C. Wriessnegger, Lena Lorenzer, Kyriaki Kostoglou

**Affiliations:** ^1^Institute of Neural Engineering, Graz University of Technology, Graz, Austria; ^2^Division of Cardiology, Medical University of Graz, Graz, Austria

**Keywords:** mental stress, EEG, ECG, mental arithmetic task, response-locked heart rate, sex differences, alpha asymmetry

## Abstract

**Introduction:**

Mental stress affects nearly everyone, with individual responses varying greatly. The importance of studying mental stress has increased, particularly during the COVID-19 pandemic. Stress has wide-ranging health impacts, from elevating blood pressure to contributing to depression and neurodegenerative conditions.

**Methods:**

This work aimed to uncover reliable correlates of mental stress using Electroencephalogram (EEG) and Electrocardiogram (ECG) methods, with an additional focus on sex differences. Twenty-five volunteers performed time-constrained mental arithmetic tasks under stress, amplified by workspace noise and negative feedback.

**Results:**

Response-locked heart rate (HR) data revealed a parasympathetic deceleration at response onset, followed by sympathetic rebound, with deeper HR dips linked to higher stress levels. Men showed earlier, longer-lasting HR decelerations, suggesting a time-based regulation strategy, while women exhibited larger, short-lived HR swings during slower responses, indicating an intensity-based response. Neural responses revealed also sex-specific stress effects: in females, stress modulated frontal theta, beta, and the theta/beta ratio–markers of cognitive control. In males, stress increased gamma and decreased delta power, indicating possibly heightened arousal and reduced motor preparation, respectively. While alpha asymmetry was modulated in both sexes, its behavioral relevance and spatial patterns differed.

**Discussion:**

These findings highlight the need for sex-specific models in neuroadaptive systems and stress-monitoring technologies.

## 1 Introduction

To understand the role of mental stress in diseases and potential treatments (Sara et al., 2022), a precise definition is essential. According to Selye, known as the “father of stress research,” stress is defined as the body’s non-specific response to any demand (Tan and Yip, 2018). Lazarus (1984) proposed the transactional stress model, describing stress as arising when a person encounters a challenging situation and is unsure how to respond. They concluded that any situation could serve as a stressor. The stress response is influenced not only by the stressor itself but also by several factors, including an individual’s perception of the stressor, their coping ability, personality type, and environmental factors (Bremner et al., 2020; Sara et al., 2022). In modern society, mental stress is prevalent in nearly everyone’s life, negatively affecting both physical and mental well-being (Esch et al., 2002; Zhang et al., 2025). The brain plays a crucial role in how the body responds to stress. The brain’s stress response is regulated by the sympathetic-adrenal-medullary axis and the hypothalamic-pituitary-adrenal axis, leading to both psychological and physiological changes (Alkadhi, 2013). Stress also induces both structural and functional changes in the brain (Yaribeygi et al., 2017). Short-term stress can increase blood pressure, weaken the immune system, and cause a decline in productivity (Al-Shargie et al., 2016). Long-term stress, on the other hand, is linked to mental disorders such as depression and is a significant risk factor for cardiovascular and neurodegenerative diseases like Alzheimer’s and Parkinson’s (Alkadhi, 2013). Given that stress affects each person differently and is a major risk factor for various diseases, a detailed understanding of its biological consequences and the identification of its correlates are necessary. There are already many tools and methods available for assessing mental stress in its early stages (Katmah et al., 2021). Psychologists often use subjective methods, such as self-reported questionnaires, to evaluate mental stress (Sara et al., 2022). Examples of these include the Self-Assessment Manikin Scale, the State-Trait Anxiety Inventory, and the Perceived Stress Questionnaire, which are commonly used in studies to assess an individual’s mental state (Halim and Rehan, 2020; Aspiotis et al., 2022; Vanhollebeke et al., 2023). However, because self-reported questionnaires rely on subjective input, they may lack accuracy (Katmah et al., 2021).

To address this issue, researchers often focus on biological and biochemical markers, such as salivary cortisol and salivary alpha-amylase levels (AlShorman et al., 2022). For instance, Al-Shargie et al. (2016) observed an increase in salivary alpha-amylase levels during stress, a finding corroborated by Hag et al. (2021), who also found elevated levels during stress conditions. Nevertheless, the accuracy of these measures can be limited by factors like the effect of physical activity on salivary alpha-amylase levels (Katmah et al., 2021). Other approaches to assessing mental stress are based on physiological factors such as heart rate (HR), skin temperature, skin conductance, respiratory rate, speech, pupil size and eye activity (de Santos Sierra et al., 2011; Hickey et al., 2021; Giannakakis et al., 2022). Among these, the most widely used method is the electrocardiogram (ECG; Hickey et al., 2021). In recent years, neuroimaging techniques have become increasingly popular for measuring mental stress with greater accuracy (Rahman et al., 2022). The most prominent methods include functional magnetic resonance imaging (fMRI), electroencephalography (EEG), functional near-infrared spectroscopy (fNIRS), and positron emission tomography (PET; Katmah et al., 2021; Vanhollebeke et al., 2022).

The methods and biomarkers used to identify mental stress vary between studies, as do the techniques employed to artificially induce stress in individuals. A common approach in many studies is the use of an arithmetic challenge to induce stress (Al-Shargie et al., 2016; Li et al., 2020; Al-Shargie, 2021; Alyan et al., 2021; Hag et al., 2021). These studies often combine mental arithmetic tasks, based on the Montreal Imaging Stress Task (MIST), with additional stressors such as time pressure or negative feedback. In a study by Ahn et al. (2019), a mental arithmetic task was combined with the Stroop Color and Word Test, a method also employed by Attar et al. (2021). In another study by Ehrhardt et al. (2022), participants completed the Paced Auditory Serial Addition Test under stress-inducing conditions, including time pressure and video recording. Vanhollebeke et al. (2023) used Raven’s Matrices with manipulated negative feedback to create stress in their participants, while Aspiotis et al. (2022) utilized virtual reality to simulate stress by exposing individuals to high altitudes.

Assessing mental stress is highly challenging, with results influenced by numerous factors. The variation in methods and types of stressors across different studies adds to this complexity.

### 1.1 EEG-based stress detection

In EEG-based stress detection, the brain region, the extracted features, and the EEG processing techniques used can lead to varying results (Katmah et al., 2021). EEG features can be categorized into time-domain features, which contain temporal information, and frequency-domain features, which represent spectral information across various frequency bands (Katmah et al., 2021). Many studies focus on the alpha, beta, and theta band powers in the prefrontal cortex (PFC) when analyzing mental stress. However, there are overlaps in the functional roles of these bands. Alpha and theta band powers are often associated with cognitive and memory performance (Klimesch, 1999). Theta band power is also known to increase during cognitive tasks such as mental arithmetic (Nigbur et al., 2011). Beta band power, linked to attention, is thought to increase during cognitive tasks and active concentration (Vanhollebeke et al., 2022). In our previous work (Wriessnegger et al., 2024), we examined how an arithmetic stress-inducing task impacted EEG. We introduced topographical maps to visualize the associations between EEG band power values in different frequency bands and several influencing factors such as task difficulty, error rate, response time, questionnaire scores, and learning effects. We observed that these factors are not independent; they overlap and collectively influence EEG activity, highlighting the complexity of interpreting EEG signals in the context of mental stress. Thus, findings related to alpha, beta, and theta band power often differ across stress-related studies. For example, Hag et al. (2021) reported decreased relative alpha band power in the PFC, along with decreased beta band power and a slight increase in theta band power during a stressful mental arithmetic task with time pressure and negative feedback. They interpreted the reduced beta power as a sign of diminished cognitive performance under stress, while the elevated theta power indicated increased cognitive effort and stress response. On the other hand, Al-Shargie et al. (2016) found a decrease in mean alpha power but an increase in mean beta power in the frontal lobe during stress. Furthermore, Halim and Rehan (2020) observed increased beta power in the parietal regions, indicating a state of heightened alertness and arousal. The findings of Al-Shargie et al. (2016) and Halim and Rehan (2020) align also with our results in Wriessnegger et al. (2024). In addition to band-specific analyses, EEG power ratios have been proposed as stress biomarkers. The gamma-to-theta (G/T) ratio, particularly in the PFC, has been suggested by Minguillon et al. (2016) 2018 as an indicator of stress during tasks like the MIST. Meanwhile, the theta-to-beta (T/B) ratio has been linked to cognitive processing capacity (Clarke et al., 2001) and shows a negative correlation with stress levels, as reported by Wen and Aris (2020) and Wen et al. (2020). These findings were also supported by our previous study in Wriessnegger et al. (2024).

Another measure related to emotions, motivation, and cognitive control is frontal alpha asymmetry (Smith et al., 2017). This asymmetry score is usually calculated by subtracting the alpha power value at F3 or F7 (left frontal electrodes) from the value at F4 or F8 (right frontal electrodes) (Vanhollebeke et al., 2022). The right hemisphere of the brain is linked to the regulation of negative emotions, while the left hemisphere is associated with positive emotion regulation (Tandle et al., 2018). As a result, increased activation of the right hemisphere (reflected by decreased alpha power relative to the left hemisphere) is expected during stress (Ocklenburg et al., 2016). Attar et al. (2021) observed an increase in frontal alpha asymmetry during stress, suggesting that the alpha power in the right hemisphere was more reduced than in the left hemisphere, indicating heightened cortical activity in the right hemisphere in response to stress. Similarly, Alyan et al. (2021) found that the right frontal hemisphere was more activated during stress compared to the left. In our previous study (Wriessnegger et al., 2024), we provided a more comprehensive view of the alpha asymmetry score, extending its analysis beyond the frontal channels to include the entire scalp. This was achieved using topographical maps to explore its relationship with stress.

### 1.2 ECG based stress detection

The most prominent physiological feature in ECG analysis is HR. During stress, HR increases due to heightened sympathetic activity, leading to a decrease in HR variability (HRV; Stauss, 2003). Vanhollebeke et al. (2023) observed an increased HR during stressful conditions, reflecting higher sympathetic activity. Many studies use ECG markers as a supplementary source alongside EEG features for detecting mental stress. For instance, Adjei et al. (2018) measured HR while participants solved mental math problems and found significantly higher HR in men compared to women. In contrast, Lucas et al. (2020) observed the opposite trend during work shifts, with women exhibiting higher HR than men. Also, Aspiotis et al. (2022) used ECG in combination with EEG to confirm whether stress was successfully induced in their participants. Hemakom et al. (2023) demonstrated that using both ECG and EEG leads to higher classification accuracy than using either alone, consistent with the findings of Ahn et al. (2019). Furthermore, (Hemakom et al., 2023) accounted for sex differences in their study, revealing that models trained separately for males and females delivered higher classification accuracy compared to mixed models. In a more recent study they observed that sex and menstrual phase differences impact stress classification performance (Hemakom et al., 2024). Therefore, considering sex differences is essential in mental stress detection research.

### 1.3 Mental stress and sex differences

It is well established that males and females exhibit different stress responses. Men typically display the “fight-or-flight” response to stress (Verma et al., 2011), while women’s reactions are additionally influenced by their menstrual cycle phase. During the follicular phase, elevated estrogen levels lead to greater parasympathetic nervous system activation, resulting in the “tend-and-befriend” response (Hemakom et al., 2023). Beyond the differences in stress responses, males and females also engage distinct brain areas under stress. In a study by Verma et al. (2011), participants completed mental arithmetic tasks during an fMRI scan. Men showed increased activation of the right frontal cortex, while women exhibited greater involvement of the limbic system. These findings align with those of Goldfarb et al. (2019), who showed participants both neutral (e.g., nature) and stressful (e.g., terror, fear) images. They found greater limbic region activation in women and higher PFC activation in men during stress. In a study using fNIRS (Zhao et al., 2021), male and female subjects solved mental arithmetic tasks of varying difficulty while researchers examined the relationship between PFC activation and performance. Men demonstrated greater PFC activation on more difficult tasks compared to women. Similarly, Kavanaugh et al. (2024) explored absolute theta band power in the mid frontal cortex of children under stress, finding that theta power was higher in girls than in boys.

Arithmetic tasks are commonly used to induce stress in research (Gärtner et al., 2015; Al-Shargie et al., 2016; Ahammed and Ahmed, 2020; Katmah et al., 2021; Wriessnegger et al., 2024). Furthermore, some studies suggest that males often outperform females in these tasks. For example, Lynn and Irwing (2008) reported that males perform better in mental arithmetic, as reflected in shorter response times and better overall performance, consistent with the findings of Yang and Yu (2021). However, Yu et al. (2024) noted that females are not inherently worse at mathematical tasks than males. They argued that social expectations may increase anxiety levels in females, leading to poorer performance. Demir and Turker (2021) focused on brain connectivity across different EEG bands to examine arithmetic performance differences between sexes. Their study found that males exhibited greater overall brain connectivity during arithmetic tasks, with notable changes in connectivity patterns across various frequency bands, particularly increased theta band connectivity in response to mental activity.

### 1.4 Motivation

Mental stress significantly affects both physical and mental well-being, potentially leading to serious conditions such as depression, cardiovascular diseases, and Alzheimer’s disease (Esch et al., 2002). To prevent stress and better understand stress-related illnesses, it is essential to thoroughly study its biological, physiological, and neurological effects. Furthermore, identifying sex-specific neural correlates of stress could pave the way for more personalized and effective prevention and treatment strategies for stress-related disorders. Understanding the underlying neurobiological differences can help in developing therapies that target the specific neural circuits and mechanisms affected by stress in men and women. This study aims to provide new insights into mental stress by identifying its neurocardiac correlates using EEG and ECG, with a particular focus on sex-related differences.

## 2 Materials and methods

### 2.1 Participants

Thirty healthy volunteers participated in this study, consisting of 16 females and 14 males, aged between 19 and 32 years. Five participants were excluded due to missing ECG signals and noisy EEG data, leaving a final sample of 25 participants, two of them were left-handed (13 females and 12 males) with a mean age of 25.08 years (standard deviation: ± 3.40). All participants were healthy adults with no history of neurological, psychiatric, or cardiovascular disorders and were not using medications affecting brain function. They had normal or corrected-to-normal vision and hearing and were fluent in the language used for the task instructions. The participants were fully informed about the experimental procedure and given ample time to ask questions. All provided voluntary written consent. During the experiment, they were instructed to sit in a relaxed position and minimize eye and body movements to prevent unwanted artifacts. The study received approval from the ethics committee of Graz University of Technology (GZ/2024).

### 2.2 Experimental paradigm

**FIGURE 1:**
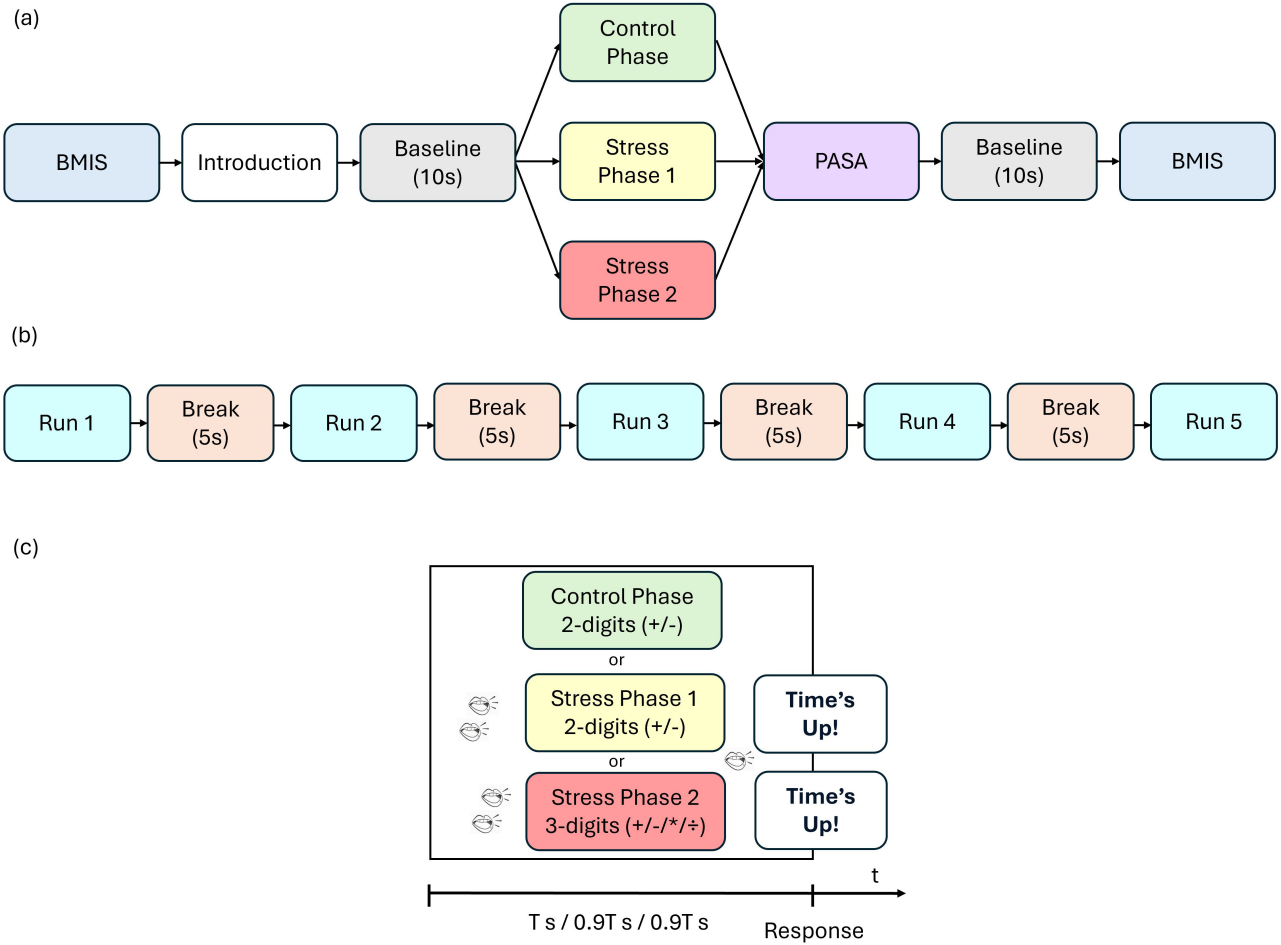
**(a)** Sequence of experimental paradigm, **(b)** Sequence of a single phase and **(c)** sequence of a single trial. Participants first completed the Brief Mood Introspection Scale (BMIS), received task instructions, and then rested for a 10 s baseline. The experiment then unfolded in three consecutive phases: a control phase, stress phase 1, and stress phase 2, followed by the Primary Appraisal Secondary Appraisal (PASA) questionnaire. A second 10 s baseline preceded the final BMIS assessment. In the control phase, participants had no time pressure (T s is assumed to be the average response time in this phase). In stress phase 1 and stress phase 2 the available time to answer was reduced by 10% compared to the average response time in the control phase (0.9 T s). Participants were also exposed to background workplace noise (such as phone ringing, laughter, or conversations) and received negative feedback for incorrect responses (“Wrong answer!”) or when exceeding the time limit (“Time’s up”).

The paradigm used in this study is based on the MIST, a commonly employed method for inducing stress in experimental conditions (Dedovic et al., 2005). At the beginning of the experiment, participants completed the Brief Mood Introspection Scale (BMIS) questionnaire (see section “2.3 Questionnaires” for more details). The experimental procedure consisted of three phases: a control phase, stress phase 1, and stress phase 2 in sequential order. In the control phase, participants were asked to solve mental arithmetic tasks without time pressure. These tasks included simple two-digit addition and subtraction (e.g., 27 + 23) as well as more complex three-digit operations involving addition, subtraction, multiplication, and division (e.g., 25 + 52 / 4). Participants indicated their answers by left-clicking the mouse on one of three possible options, with the range of potential solutions limited to values between 0 and 99. Stress phase 1 involved only two-digit addition and subtraction problems, but the available time to answer was reduced by 10% compared to each participant’s average response time in the control phase. To heighten the stress experienced, participants were exposed to background workspace noise (e.g., phone ringing, laughter, or conversation) while solving the mathematical problems. Additionally, they received negative feedback for incorrect answers (“Wrong answer!”) or when the time limit was reached (“Time’s up”). Stress phase 2 was generally similar to stress phase 1, except that it utilized advanced three-digit calculations. Each phase included five runs, each consisting of 15 trials, with a 5-s break between runs during which participants focused on a fixation cross. This resulted in a total of 225 trials per participant. A baseline measurement was recorded for 10 s before each phase and after the final phase, during which participants again focused on a fixation cross. Additionally, after each phase, participants completed the Primary Appraisal Secondary Appraisal (PASA) questionnaire (see section “2.3 Questionnaires” for more details) to assess their self-reported stress levels, taking approximately 1–2 min. The experiment concluded with a second BMIS questionnaire. Figure 1 illustrates the complete experimental sequence (Figure 1a) and the details of a single phase (Figure 1b).

### 2.3 Questionnaires

The BMIS was used to assess the participants mood before and after the experiment. Mayer and Gaschke (1988) developed the BMIS questionnaire, which comprises 16 adjectives describing various moods (Table 1). Participants rated each adjective on a four-point scale from 1 (“*definitely do not feel*”l) to 4 (“*definitely feel*”). The BMIS score is obtained by subtracting the total (i.e., sum) score of the negative adjectives from the total score of the positive adjectives. Additionally, there is a scale to rate overall mood, ranging from −10 (very unpleasant) to 10 (very pleasant). The BMIS questionnaire is frequently used to assess changes in an individual’s feelings before and after a specific situation.

**TABLE 1:** BMIS adjectives used to describe the emotional state of the participant. The scale was defined as follows: a score of 1 indicated “*definitely do not feel,*” 2 corresponded to “*do not feel,*” 3 represented “*slightly feel*” and 4 signified “*definitely feel.*”

Positive adjectives	Negative adjectives
Happy	Sad
Lively	Tired
Caring	Jittery
Content	Drowsy
Peppy	Fed up
Loving	Gloomy
Active	Grouchy
Calm	Nervous

The PASA questionnaire was used to assess self-experienced stress after control, stress 1 and stress 2 phase (Gaab, 2009). It is based on the transactional stress model of Lazarus (Lazarus, 1984) and used for the self-assessment of situation-specific stress. It includes 16 statements, whereby each statement can be rated from 1 (completely wrong) to 6 (completely right) points (Table 2). The measure consists of the four primary scales: threat, challenge, self-concept of own competence, and control expectancy (Table 2). Combining the primary scales threat and challenge results in the primary appraisal. The secondary appraisal is computed with the primary scales self-concept of own competence and control expectancy. The primary appraisal scale assesses the relevance of a situation to an individual’s well-being and the secondary appraisal scale reflects an individual’s perceived resources to cope with a stressor. Finally, the global PASA index can be calculated by the difference between the primary and secondary appraisal scales, which determines the transactional stress perception of an individual person. A lower PASA index score can be interpreted as low cognitive stress level.

**TABLE 2:** PASA items for different subscales. Note that items marked with an asterisk were reverse-scored (i.e., assigned a value of 7 minus the original score), as they convey the opposite meaning and effect compared to the other items within the same category. The scale ranged from 1 to 6, where 1 corresponded to “*completely wrong,*” 2 to “*mostly wrong,*” 3 to “*somewhat wrong,*” 4 to “*somewhat right,*” 5 to “*mostly right,*” and 6 to “*completely right.*”

Category	Subcategory	Questions
Primary appraisal	*Threat*	I do not feel threatened by the situation*
		This situation is very uncomfortable for me
		I do not feel worried because the situation does not pose a threat to me*
		This situation scares me
	*Challenge*	The situation is important to me
		I do not care about this situation*
		The situation is not a challenge for me*
		This situation challenges me
Secondary appraisal	*Self-efficacy*	In this situation, I know what I can do
		I have no idea what I should do now*
		In this situation, I can think of many possible courses of action
		I can think of many solutions for this situation
	*Control expectancy*	It mainly depends on me whether I can cope with the situation
		I can best protect myself against failure through my own behavior
		I can control a lot of what happens in this situation myself
		If I overcome this situation, it is the result of my effort and my personal commitment

### 2.4 Data acquisition

The complete paradigm, including presenting arithmetic tasks for control phase, stress phase 1 and stress phase 2 as well as the PASA questionnaire, was designed with the Python toolbox PsychoPy. The time markers were also implemented with PsychoPy. To synchronize all signals from the paradigm and the recording devices, the Lab Streaming Layer software was used. For the EEG data acquisition, the mobile, active EEG system LiveAmp from Brain Products (Gilching, Germany) was used in combination with the actiCAP electrode cap. A total of 32 active electrodes were mounted according to the international 10–20 system (FP1, FP2, F3, F4, Fz, F7, F8, FC1, FC2, FC5, FC6, FT9, FT10, C3, C4, CZ, T7, T8, CP1, CP2, CP5, CP6, TP9, TP10, P3, P4, P7, P8, PZ, O1, O2, OZ). FCz was used as the reference electrode and FPz as the ground electrode. The sampling frequency of the EEG recordings was 500 Hz.

For the first three participants the connectible box of the LiveAmp was used for ECG signal recording. It simultaneously recorded EEG and ECG signals using a sampling frequency of 500 Hz. Due to problems with the connectible box of the LiveAmp the g.USBamp (Gtec medical engineering, Austria) was used to record ECG for the rest of the participants with a sampling frequency of 256 Hz. The setup used was a custom bipolar ECG configuration, with the ground electrode placed on the left side on the last ribcage and the cathode and anode directly below the collarbone symmetric on the right and left side, respectively.

### 2.5 Signal preprocessing

Signal preprocessing was performed in MATLAB 2024b, Mathworks. A zero-phase, fourth-order Butterworth IIR filter was applied to bandpass filter the EEG and ECG signals between 0.5 and 60 Hz. To attenuate power line interference at 50 Hz, a second-order Butterworth IIR notch filter was used. To exclude unwanted artifacts from the EEG signals, independent component analysis (ICA) was performed using the EEGLAB toolbox v.2023.0 (Delorme and Makeig, 2004) with the default extended Infomax algorithm (*runica*) (Lee et al., 1999). No channel interpolation, rejection, or re-referencing was performed before applying ICA. To assist in identifying components reflecting artifacts such as eye movements, muscle activity, or noisy channels, we used the ICLabel (Pion-Tonachini et al., 2019) plugin as a guidance tool. However, final decisions on component rejection were based on careful visual inspection. Only components clearly identified as non-neural, whether due to physiological artifacts or channel noise, were removed (typically ranging from 1 to 5 components per participant). The HR signal was derived from the ECG using the Pan-Tompkins algorithm (Pan and Tompkins, 1985; Sedghamiz, 2025). Once the R-peaks were identified, HR was computed as the reciprocal of the R-R intervals (i.e., 1 / R-R interval), then multiplied by 60 to express the result in beats per minute (bpm). Prior to that, however, we identified and removed artifacts based on abrupt, physiologically implausible changes between successive heartbeats. After this, we manually corrected the cleaned R-R series by identifying remaining outliers using the generalized extreme studentized deviate (GESD) method (Rosner, 1983), implemented via MATLAB’s *filloutliers* function. The GESD approach is designed to detect multiple statistical outliers in a dataset, even when they occur in clusters or deviate strongly from normality. Detected outliers were then replaced using linear interpolation. Finally, any remaining missing values were filled using standard linear interpolation to produce a continuous, artifact-free R-R time series. Since R-R intervals are inherently non-uniformly sampled, the resulting HR signal was linearly interpolated and upsampled to match the EEG sampling rate, enabling synchronized analysis. We acknowledge that differences in ECG acquisition systems can introduce variability in signal characteristics, even with consistent electrode placement. However, our analysis focused on HR rather than detailed ECG waveform morphology. Prior to HR extraction, we visually inspected all ECG recordings to ensure signal quality and found them to be sufficiently clean for reliable R-peak detection and R–R interval estimation across participants. For subsequent analysis, the HR signal was also baseline-corrected per phase by computing the HR change from the baseline period immediately preceding each phase, thereby accounting for individual differences in resting HR and emphasizing task-evoked physiological reactivity. This procedure effectively removes any slow drifts or baseline shifts in HR over the course of the experiment, ensuring that all reported effects reflect true task-related changes. To ensure consistency across participants despite differences in recording systems and sampling frequencies, all signals were ultimately resampled to 256 Hz using MATLAB’s resample function, which applies an anti-aliasing filter prior to downsampling.

All EEG signals were subsequently segmented into trials centered on each participant’s response time, covering a window from −3 to −0.5 s relative to the response. This alignment was chosen to capture the neural and physiological processes leading up to the behavioral response, such as attentional engagement, motor preparation, and autonomic adjustments, while avoiding the influence of early sensory components such as visual-evoked potentials triggered by the initial presentation of the mathematical problems. Aligning to the response, rather than the stimulus, also minimized variability due to individual differences in reaction time, enabling more accurate comparisons across participants and conditions. EEG trials exhibiting excessive noise were excluded based on amplitude thresholding at ± 100 μV. On average, 2.23 ± 2.23 trials per participant were discarded. For HR analysis, we used a time window from –4 to + 6 s relative to the response, allowing us to capture both pre-response and post-response dynamics.

### 2.6 Feature extraction

For each epoched EEG trial, we extracted spectral features across five standard frequency bands: delta (D; 0.5–3.5 Hz), theta (T; 4–7.5 Hz), alpha (A; 8–12.5 Hz), beta (B; 13–30 Hz), and gamma (G; 30–60 Hz). Band power within each range was computed using MATLAB’s *bandpower* function. Because our 10-s pre-phase resting segments are too brief and contaminated by anticipatory arousal to provide a stable reference, we opted not to baseline correct the EEG band power values. Instead, similarly to our previous work (Wriessnegger et al., 2024), to reduce inter-subject and inter-channel variability, we calculated relative band power by dividing the absolute power in each band by the total power summed across the broad (0.5–60 Hz) band within each trial. In this way, spectral power was normalized on a per-trial basis, allowing us to focus on the relative distribution of power across frequency bands. Moreover, given our cumulative stress design, imposing a single “fixed” EEG baseline at the start would obscure the progressive, phase-by-phase buildup of stress.

In addition to relative power, we computed the T/B and G/T power ratios, which serve as established indicators of cognitive and attentional states (Clarke et al., 2001; Arns et al., 2013; Putman et al., 2014; Angelidis et al., 2016; Minguillon et al., 2016, 2018; Wen and Aris, 2020; Wen et al., 2020; Zivan et al., 2023). We also examined hemispheric asymmetries in the alpha band by calculating laterality indices: ln(L)-ln(R) for left hemisphere channels and ln(R)-ln(L) for right hemisphere channels, where L and R denote relative power. This approach captures shifts in hemispheric dominance, with increases in one hemisphere mirrored by reductions in the other. Importantly, using relative power to estimate laterality indices helps mitigate the impact of referencing artifacts and global amplitude fluctuations. In (Vincent et al., 2021) frontal asymmetry estimates based on relative power were found to provide more reliable and interpretable results compared to those based on absolute power measures. Thus, here we used the relative power to estimate laterality indices. While asymmetry analyses typically focus on predefined electrode pairs—especially in frontal regions—our aim was to capture more global lateralization effects. To this end, we performed a whole-head analysis by computing the laterality index across all homologous electrode pairs.

### 2.7 Error rates and response times

For each participant, the error rate (%) was calculated as the proportion of incorrect responses within each run (comprising 15 trials) and task phase. Response time was measured in seconds. If a participant did not provide a response, the trial was treated as an error, and the maximum allotted time for that subject and phase was assigned as the response time. We also computed relative response times defined as each subject’s response time divided by the average response time of all participants for the respective phase across all runs, in order to account for phase-related differences in maximum allotted response times.

### 2.8 Linear mixed effects models

Building on our previous study (Wriessnegger et al., 2024), we employed linear mixed-effects (LME) models to investigate the relationships between variables (MATLAB function *fitlme*). The model structure used was: Y ∼ X + (1| Participant), which includes a random intercept for each participant to account for individual baseline variability—such as differences in EEG power. This formulation captures both fixed effects of the predictor (X) and random effects due to participant-level differences. Consistent with our prior findings (Wriessnegger et al., 2024), we observed that including a random intercept substantially improved model fit, as evidenced by lower Akaike information criterion (AIC) values when compared to models that excluded this random effect. Model parameters were estimated using maximum likelihood. The strength and direction of the relationship between X and the outcome variable Y were evaluated using the *t*-statistic of the fixed effect, defined as the estimated coefficient divided by its standard error. Positive or negative *t*-values reflect the nature of the association.

The variables of interest included EEG band power across five frequency ranges (D, T, A, B, G), T/B and G/T ratios, and laterality scores for the A band. These features were examined in relation to a set of predictors: experimental phase (i.e., control phase, stress phase 1 and stress phase 2), error rate and stress ratings derived from the PASA questionnaires (administered after each phase). For each model, the dependent variable (Y) represented the average EEG measure across each run at a specific electrode, aggregated across all participants, while the predictor (X) corresponded to one of the aforementioned factors. Fixed-effect *t*-values from each channel were extracted and compiled into spatial representations—referred to as *t-maps*—to visualize how EEG features relate to each factor.

### 2.9 Statistical analysis

In the LME models, degrees of freedom for fixed effects were computed using the residual method, defined as the number of observations minus the number of fixed-effect parameters, and two-tailed *p*-values were obtained from the corresponding *t*-distribution, as implemented in MATLAB’s *fitlme* function. Significant effects across EEG channels in the t-maps were determined by applying the Benjamini-Hochberg (Benjamini and Hochberg, 1995) procedure to control for multiple comparisons on the *p*-values obtained from the LME models. To assess differences between males and females, we employed the Wilcoxon rank-sum test for questionnaire responses, error rates, response times, and HRs. For within-group comparisons (i.e., within the same sex), we used the Wilcoxon signed-rank test. Note that the Benjamini-Hochberg correction was applied consistently in all analyses involving multiple statistical tests. For all tests based on Wilcoxon tests (i.e., rank-sum and signed-rank), we also computed effect sizes using Cohen’s *r*, calculated by dividing the standardized test statistic (Z) by the square root of the total number of observations, providing a consistent measure of effect magnitude across both independent and paired comparisons. For LME models, we extracted each fixed-effect term’s *t*-statistic (*t*) and its associated degrees of freedom (*df*). We then converted these into Cohen’s *r* using the formula $r\,s\,i\,g\,n\,\left(t\right)\,\sqrt{\frac{t^{2}}{t^{2}+d\,f}}$ (Fritz et al., 2012), which yields a signed, unit-less measure of effect magnitude (–1 to + 1). This approach provides an effect-size metric directly comparable to the Wilcoxon-test *r*, interpretable with the same benchmarks (≈0.1 small, ≈0.3 medium, ≥0.5 large), and allows standardized comparison across both mixed-model predictors and non-parametric tests.

## 3 Results

### 3.1 Behavioral results

**FIGURE 2:**
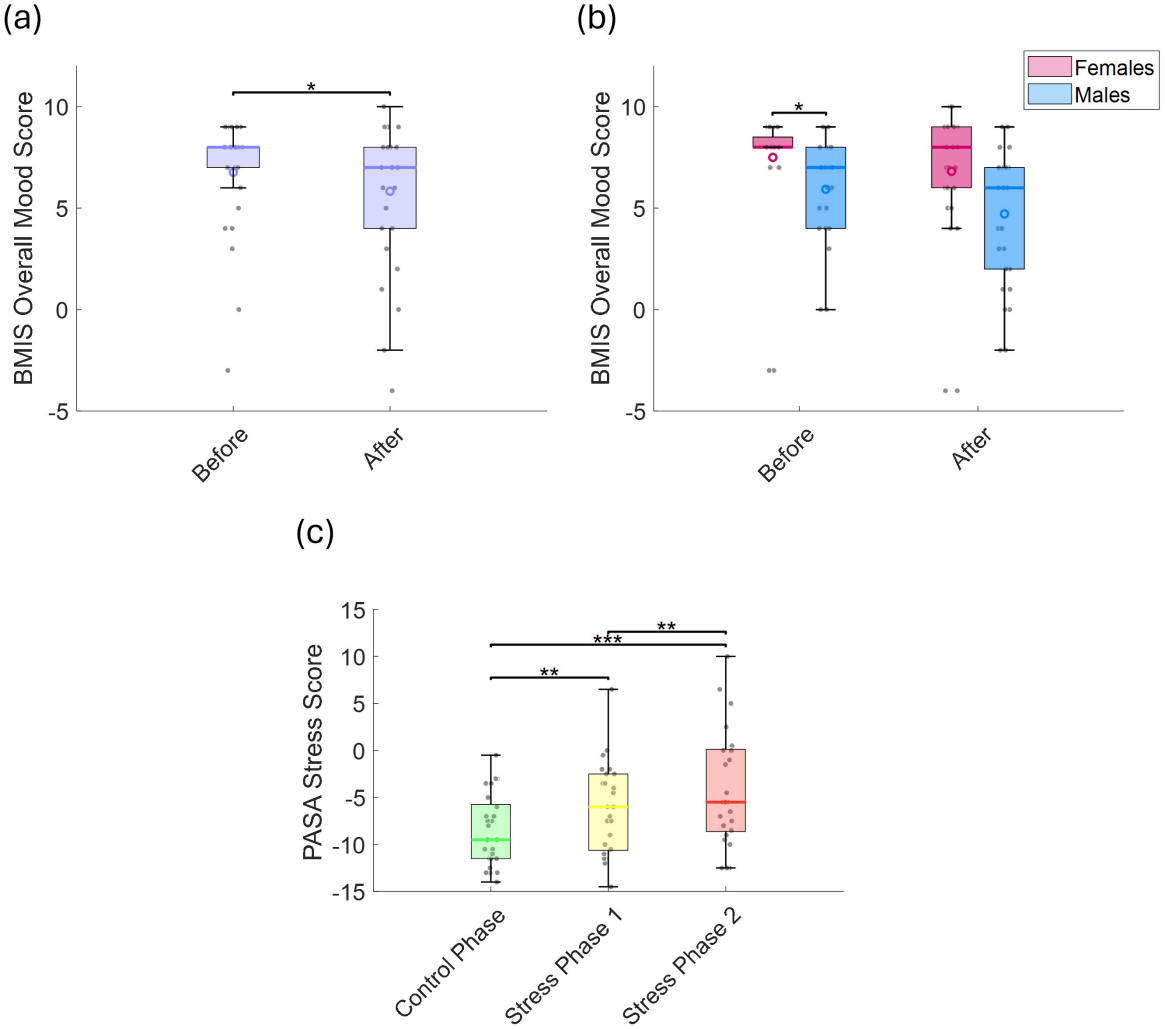
**(a)** BMIS overall mood scores before and after the experiment, and **(b)** comparison between sexes. **(c)** PASA stress scores across the control phase, stress phase 1, and stress phase 2. Statistically significant differences between conditions are indicated by asterisks (*p < 0.05, **p < 0.01, ***p < 0.001).

We compared BMIS and overall mood scores before and after the experiment. The BMIS mood score significantly decreased after the experiment *(p* = 0.025, *r* = 0.631; Figure 2a), indicating a shift toward more negative feelings. No general sex-related differences were observed (Figure 2b). However, males reported significantly lower overall mood scores than females prior to the experiment (*p* = 0.039, *r* = 0.513; Figure 2b). Similarly, PASA stress scores—which reflect perceived stress levels—increased significantly from the control phase to stress phase 1 (*p* = 0.014, *r* = 0.642) and stress phase 2 (*p* < 0.001, *r* = 0.657) (Figure 2c), with no significant sex differences observed.

**FIGURE 3:**
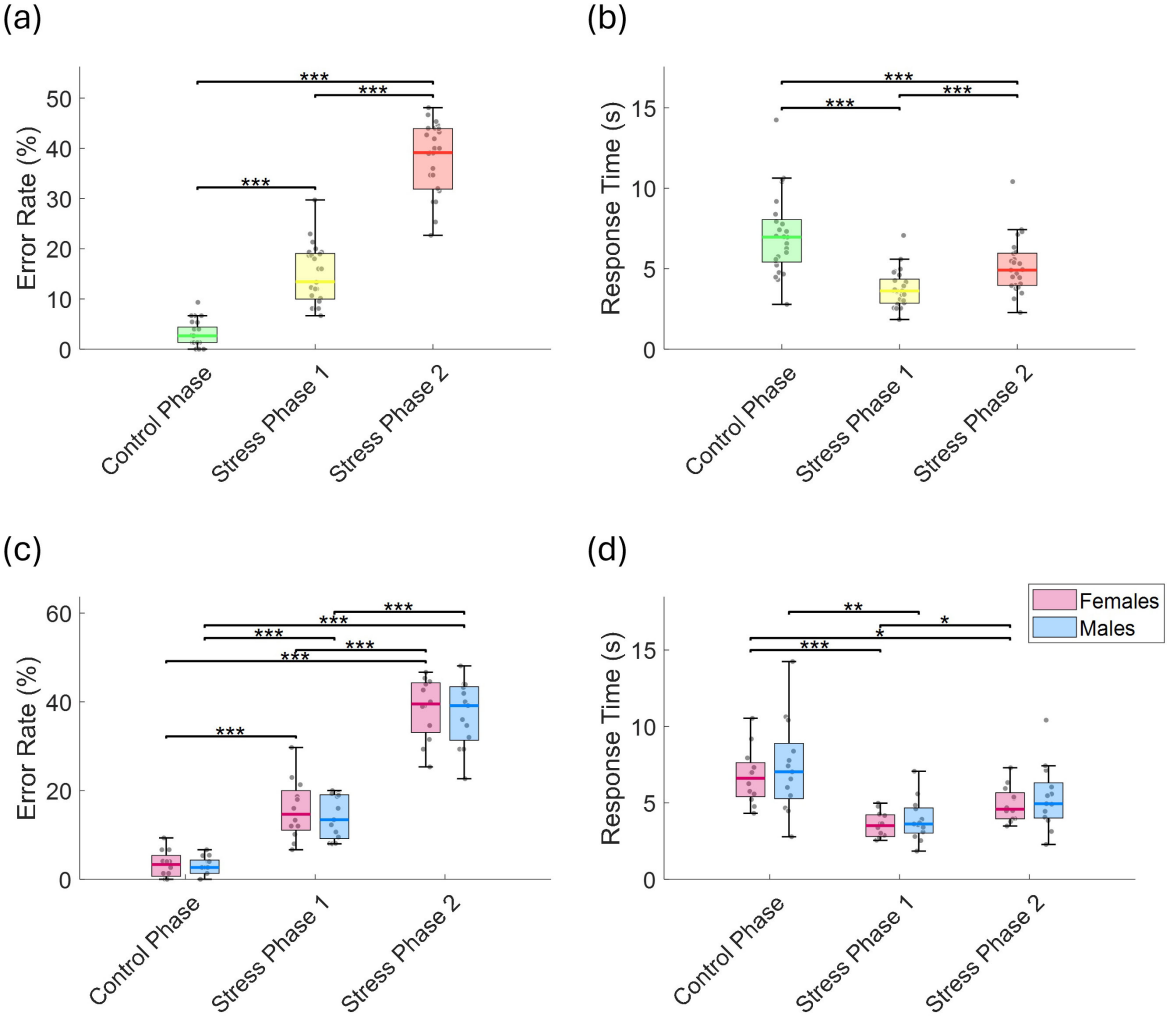
**(a)** Error rate (%), and **(b)** response time (s) across the control phase, stress phase 1, and stress phase 2. **(c)** Error rate (%), and **(d)** response time (s), separated by sex. Statistically significant differences between conditions are indicated by asterisks (*p < 0.05, **p < 0.01, ***p < 0.001).

The mean error rate increased significantly across phases: from 3.1 ± 2.5% in the control phase to 14.8 ± 5.7% in stress phase 1 (*p* < 0.001, *r* = 0.873), and further to 37.8 ± 7.0% in stress phase 2 (*p* < 0.001, *r* = 0.873; Figure 3a). Mean response time in the control phase was 7.0 ± 2.5 s, which significantly decreased to 3.7 ± 1.1 s in stress phase 1 (*p* < 0.001, *r* = 0.347), and then significantly increased again to 5.1 ± 1.6 s in stress phase 2 compared to stress phase 1 (*p* < 0.001, *r* = 0.873; Figure 3b). This corresponds to total durations of approximately 8.8 min for the control phase, 4.6 min for stress phase 1, and 6.4 min for stress phase 2. These estimates exclude the 5-s inter-run fixation breaks and the time required for completing the PASA questionnaire after each phase. No significant differences were found between sexes in either error rate or response time (Figures 3c, d).

**FIGURE 4:**
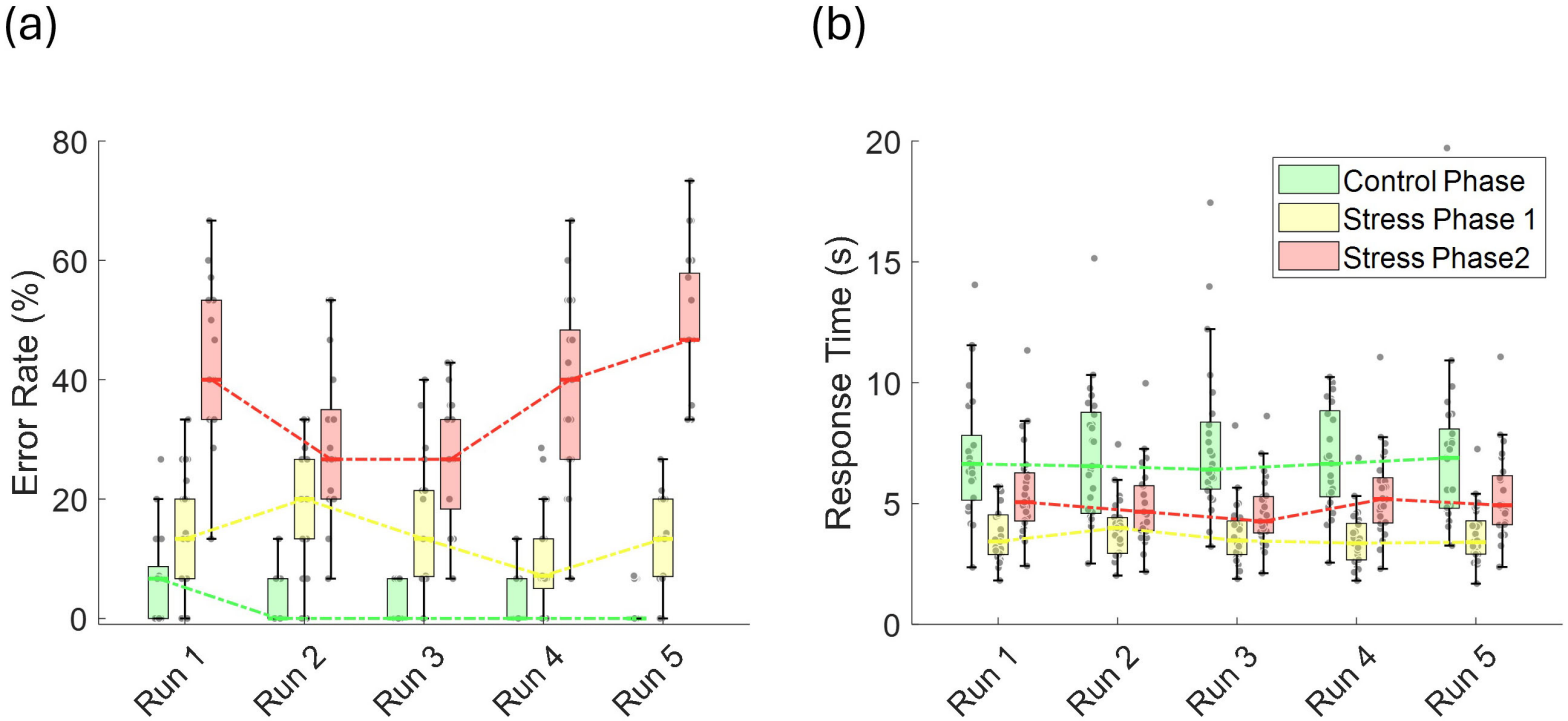
**(a)** Error rate (%), and **(b)** response time (s) across runs for the control phase, stress phase 1, and stress phase 2.

To assess potential practice-related improvements, we analyzed trends in error rates and response times across repeated task runs. LME models were fitted separately for each phase, with run number as the predictor and either error rate or response time as the dependent variable. During the control phase, error rates showed a significant linear decline (*t* = −4.59, *p* = 1.06 × 10^–5^, *r* = 0.380), indicating improvement over time (Figure 4a). In stress phase 1, although a downward trend was observed, the change was not statistically significant decline (*t* = −1.813, *p* = 0.072, *r* = 0.161). Interestingly, in stress phase 2, a significant time effect emerged (*t* = 2.29, *p* = 0.023, *r* = 0.201), characterized by a drop in error rate up to run 3, followed by an increase through run 5. No significant run-related effects were observed for response times in any phase (Figure 4b), and there were no sex-related differences in the patterns of error rates or response times.

### 3.2 ECG results

To capture task-evoked autonomic dynamics, HR was time-locked to response onset (*t* = 0 s), a behavioral marker reflecting decision or motor action. This alignment allows precise comparisons of anticipatory, reactive, and recovery-related cardiovascular changes across conditions and participants.

**FIGURE 5:**
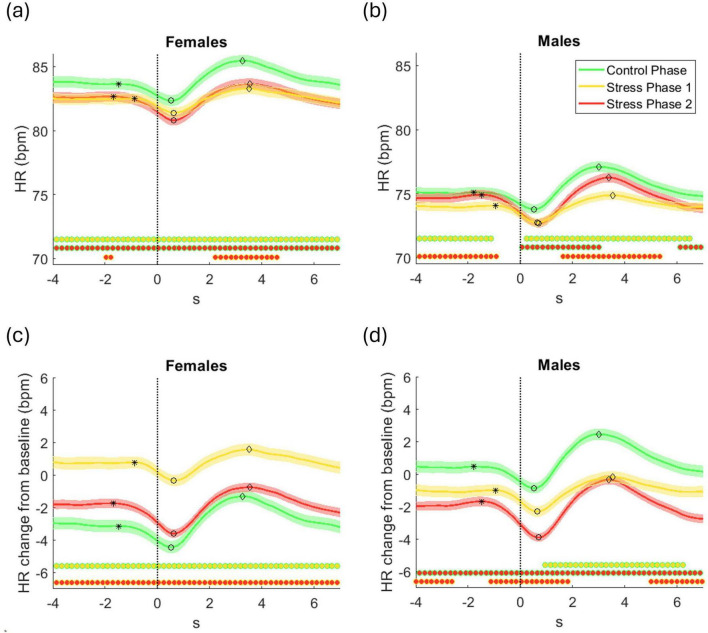
Grand average ( ± standard error) across all participants of **(a,b)** HR in beats per minute (bpm), and **(c,d)** HR change relative to baseline (bpm), time-locked to the response onset (*t* = 0 s), for the control phase, stress phase 1, and stress phase 2 for **(a,c)** females and **(b,d)** males. Statistically significant differences between phases at each time point are indicated by filled circles along the *x*-axis (*p* < 0.05, corrected for multiple comparisons). Each cluster comparison is color-coded, with the outer edge and inner fill colors denoting the corresponding phase comparison (e.g., green-edged circles with a yellow fill indicate significant differences between the control phase and stress phase 1). Asterisk (*), circle (○), and diamond (⋄) markers on each HR trajectory indicate the HR dip onset time, the time of the HR minimum (dip), and the HR rebound time, respectively.

Figure 5 shows grand average HR responses time-locked to response onset for three phases–control, stress 1, and stress 2–presented in bpm and as changes from baseline (herein referred to as baseline-corrected HR). Data are separated by sex: females in Figures 5a, c and males in Figures 5b, d. Significant phase differences at each time point are marked by filled circles, with edge/fill color indicating the comparison.

Raw and baseline-corrected HR captured complementary aspects of stress responses. The sequential design (control → stress 1 → stress 2) means raw HR reflects both immediate stress effects and cumulative physiological changes (e.g., fatigue or adaptation). In all participants, HR dipped around response onset, then rebounded—indicating parasympathetic deceleration followed by sympathetic activation

Females (Figure 5a) showed higher raw HR than males, with HR peaking in control and dropping in both stress phases. Post-dip HR stayed elevated in control but remained suppressed in stress phases. Differences between control and each stress phase were significant; subtler distinctions emerged between the two stress levels. Males (Figure 5b) showed a more graded pattern: highest HR in control, lowest in stress 1, intermediate in stress 2. Post-dip recovery followed the same order, suggesting a stepwise modulation by stress and time-on-task.

Baseline-corrected HR isolated within-phase changes that were less apparent in raw data. In females (Figure 5c), this approach clarified the stress effects: although raw HR looked similar across stress phases, baseline-corrected data revealed a significant HR increase above baseline in stress phase 1, while both control and stress phase 2 showed suppression–most strongly in the latter. These patterns indicate enhanced HR reactivity under moderate stress and reduced responsiveness under high stress. In males (Figure 5d), baseline-corrected HR revealed a more linear trend. HR was elevated above baseline only during the control phase, whereas both stress phases showed reductions, with the strongest suppression in stress phase 2. These graded decreases suggest a monotonic decline in autonomic reactivity as stress intensity increases.

The dynamics of the HR dip and rebound varied across experimental phases and between sexes. To quantify these dynamics, several features were extracted from the average baseline-corrected HR trajectories, computed separately for each participant and phase. The *HR dip onset* was identified as the first point before the HR minimum where the HR velocity became negative following a period of near-zero change (Figure 5). The *HR dip magnitude* was defined as the difference between the HR at onset and the minimum HR value, and the *HR dip timing* corresponded to the time of this minimum. The *HR dip duration* was the interval from onset to minimum. The *HR rebound magnitude* was the difference between the minimum HR and the subsequent local maximum, with *HR rebound timing* and *HR rebound duration* referring to the time and interval from the minimum to this peak, respectively.

To statistically assess these changes and identify the contributing factors, we applied linear regression models of the following form in Wilkinson notation: Y ∼ sex*X, where Y represents one of the previously defined baseline-corrected HR trajectory features, estimated from the average responses of each participant and X represents one of the following predictors: error rate, PASA stress score, task phase, or relative response time. This formulation expands to include both the main effects of each variable and their interaction with sex. Error rate and response time were computed as the participant’s total values for each respective phase. The variable phase was treated as a categorical factor coded as 1 (control), 2 (stress 1), and 3 (stress 2), while sex was coded as 1 for females and 2 for males.

**FIGURE 6:**
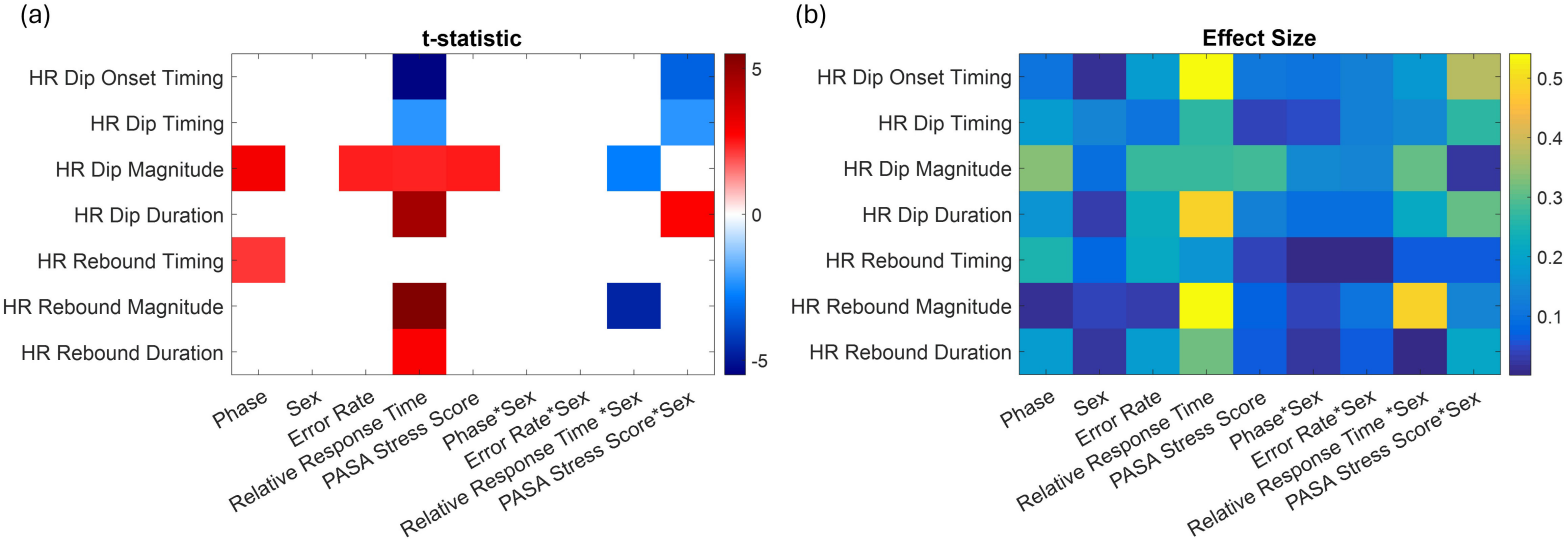
**(a)** Significant predictors of baseline-corrected HR response metrics based on linear models. The heatmap displays *t*-statistics for predictors (*x*-axis) significantly associated with different HR features (*y*-axis), including dip timing, magnitude, onset, rebound timing, and rebound magnitude. Only predictors with *p* < 0.05 (corrected for multiple comparisons) are shown. Red colors indicate positive associations, and blue colors indicate negative associations. Significant interaction terms involving sex highlight sex-dependent effects and how the influence of other predictors varies between females and males. **(b)** Corresponding effect sizes (Cohen’s r).

Figure 6 shows, in panel (a), a heatmap of predictors that reached statistical significance (*p* < 0.05), and in panel (b), the corresponding effect sizes. These panels summarize the outcomes of linear regression models assessing how each factor impacts various HR response features. The rows correspond to specific HR features while the columns represent significant main effects and interaction terms. The color scale reflects the *t*-statistics of each effect, with red colors indicating positive associations and blue colors indicating negative associations.

Several key patterns emerge from this analysis. Relative response time was significantly associated with multiple HR features: individuals with slower responses exhibited earlier HR dip onset and dip timing, longer dip duration, and greater rebound magnitude and duration, indicating that delayed behavioral responses were linked to earlier and stronger autonomic reactivity. PASA stress scores were positively associated with HR dip magnitude, suggesting that higher stress was linked to larger initial HR drops. HR dip magnitude increased from control to stress phases and rebound timing was delayed. Significant sex interactions highlighted sex-specific differences in the temporal dynamics of HR regulation. For example, in response to increased stress, males showed an earlier onset and timing of the HR dip, along with a longer dip duration compared to females (PASA * Sex). In contrast, females exhibited a delayed HR dip with a shorter duration. Additionally, women demonstrated greater HR dip and rebound magnitudes associated with longer response times (Response Time * Sex).

### 3.3 EEG results

**FIGURE 7:**
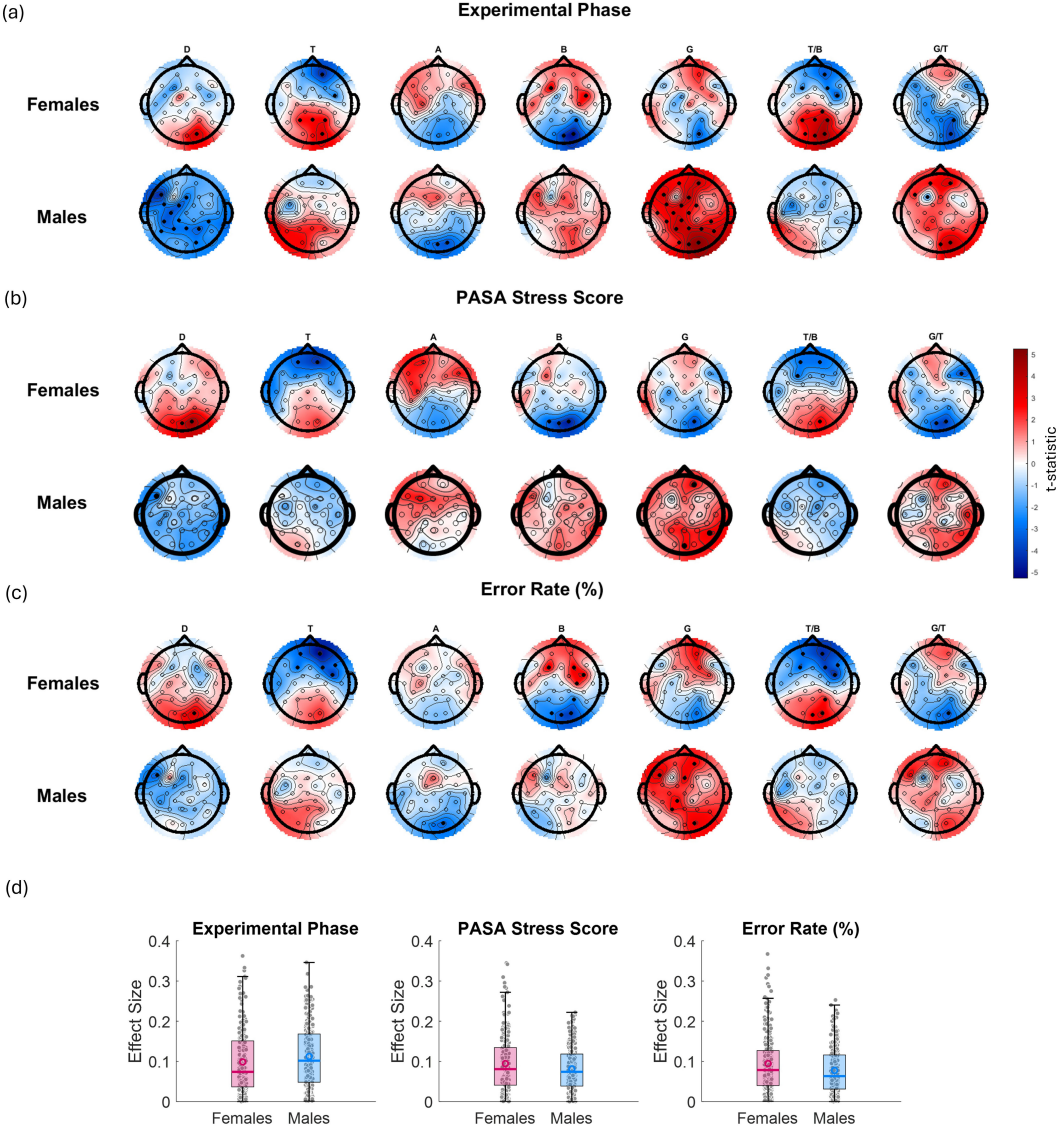
*t*-maps showing the relationships between EEG features (from left to right: D power, T power, A power, B power, G power, T/B ratio, and G/T ratio) and various variables: **(a)** experimental phase (as 1 for control, 2 for stress phase 1, and 3 for stress phase 2—red shading indicates an increase in the EEG feature from control phase to stress phase 2, while blue indicates a decrease), **(b)** PASA stress scores (from questionnaires administered after each phase) and **(c)** error rate (%) for males and females separately. Red gradients denote positive associations, while blue gradients indicate negative associations. Channels showing statistically significant effects (*p* < 0.05) are marked with filled black circles. **(d)** presents boxplots of the corresponding effect sizes (Cohen’s r) calculated across all channels and bands for each variable and sex.

In Figure 7, each t-map shows the spatial distribution of associations (*t*-values) between a predictor variable (experimental phase, PASA stress score, error rate) and EEG features (columns: D, T, A, B, G power, and the T/B and G/T ratios) for females and males separately. Red indicates a positive relationship; blue, a negative one. Statistically significant associations (*p* < 0.05) within channels are marked by filled black circles. Effect sizes are also provided (Figure 7d). Across all three predictors, highly similar spatial and spectral patterns of EEG modulation were observed in both sexes. These consistent effects suggest that all three factors likely index a common underlying process, such as stress-induced cognitive load or mental effort.

In Figure 7a, which reflects the effect of experimental phase (from control to stress phase 2), females showed significant negative associations with T power at frontal electrodes and corresponding reductions in the T/B ratio (i.e., T frontal power and T/B ratio decreased as the experiment progressed from control to stress phase 2). In addition, B power significantly increased at right parietal electrodes, indicating a localized shift toward higher-frequency activity under stress. In males, although similar overall patterns were observed, the most statistically significant effects were found in the D and G bands. Specifically, D power significantly decreased over central and parietal electrodes especially on the left hemisphere, while G power showed widespread and robust increases across posterior and parietal regions. These changes were accompanied by significant increases in the frontal G/T ratio. In Figure 7b, relating EEG features to PASA stress scores, females showed significant negative associations with frontal T power and decreased frontal T/B ratios. Males again displayed significant increases in G power. In Figure 7c, examining error rate, revealed that females displayed significant reductions in T power and increases in B power at frontal electrodes, as well as decreased T/B ratios over mid-frontal sites with increasing error rates. In males, significant widespread increases in G power, especially across posterior electrodes were observed. Taken together, these results show that while both sexes exhibit stress- and performance-related modulation of EEG features, D and G band power changes—particularly G—emerge as the most consistently significant in males, while T power, B power, and T/B ratio show stronger and more localized effects in females.

**FIGURE 8:**
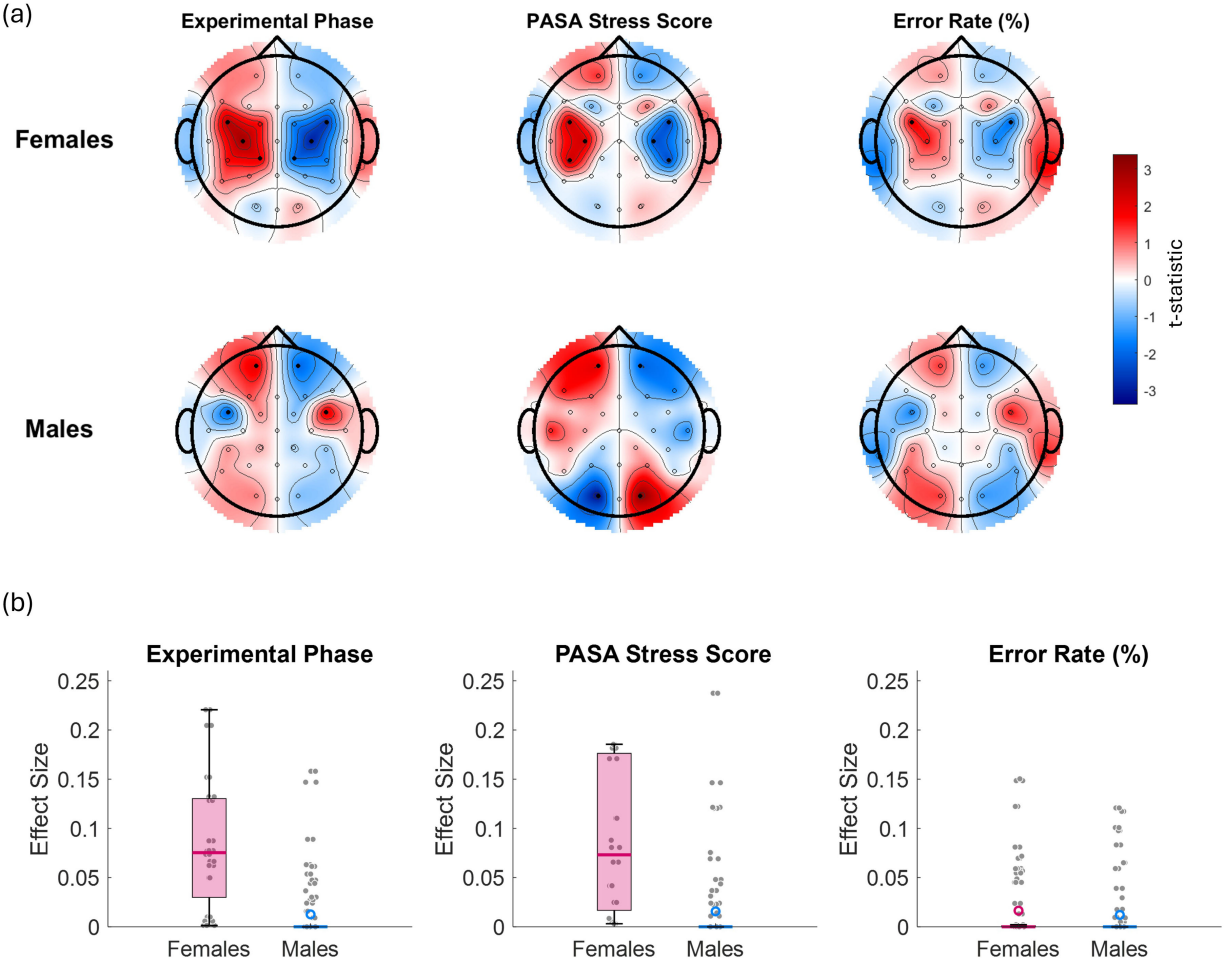
**(a)** *t*-maps illustrating the relationship between alpha asymmetry and the experimental phase (coded as 1 for control, 2 for stress phase 1, and 3 for stress phase 2), PASA stress scores, and error rate (%) separately for females (top panel) and males (bottom panel). Red regions indicate areas where laterality power scores increase with increasing values of the corresponding variable, while blue regions reflect a decrease. Laterality values along the midline were set to zero. Statistically significant channel effects (*p* < 0.05) are denoted by solid black dots. **(b)** presents boxplots of the corresponding effect sizes (Cohen’s r) calculated across all channels for each variable and sex.

Figure 8 illustrates the relationship between alpha laterality and three key variables—experimental phase, PASA stress score, and error rate—displayed separately for females (top row) and males (bottom row). Red regions indicate increased laterality values (i.e., greater relative left- or right-hemisphere alpha power dominance depending on the side), while blue regions reflect decreased laterality. Statistically significant effects (*p* < 0.05) are denoted by solid black dots. In females, significant effects were observed over left and right frontocentral and centroparietal electrodes for both the experimental phase and PASA stress scores, indicating that A asymmetry in these regions reliably tracked increases in task-induced stress and self-reported stress. Additionally, for error rate, a significant effect was observed over frontocentral channels (and specifically FC5/FC6), suggesting that increased error rates were associated with a shift in frontal A asymmetry. In males, significant effects for experimental phase were found over frontal (FP1/FP2) and frontocentral electrodes (FC5/FC6), while PASA stress scores showed significant associations with frontal (FP1/FP2) and occipital (O1/O2) regions. For error rate, no significant effects were observed.

Visual comparison of the t-maps in Figure 8 reveals that the experimental phase effects closely resemble a combination of the PASA stress score and error rate maps. This suggests that experimental phase effects largely reflect a composite of increasing subjective stress and accumulating performance variability across phases, rather than an independent source of EEG asymmetry modulation.

## 4 Discussion

The primary objective of this study was to identify reliable indicators of mental stress using EEG and ECG features. Well-established indicators were employed based on prior research (Attar et al., 2021; Hag et al., 2021; AlShorman et al., 2022; Aspiotis et al., 2022). In addition to these objective measures, subjective experiences of mood and perceived stress were assessed using two validated questionnaires: the BMIS and the PASA scale. A key objective of the study was also to investigate sex-related differences in the neurophysiological and behavioral responses to stress. The following discussion integrates findings from the EEG, ECG, and questionnaire data, with an emphasis on how stress responses may differ between males and females across multiple domains.

### 4.1 Subjective and behavioral indicators of stress

The behavioral and self-report data provide clear evidence that the experimental manipulation successfully induced psychological stress and impacted task performance. Participants’ mood, as assessed by the BMIS, declined significantly following the experiment, indicating a shift toward more negative affective states. This supports the notion that the cognitive load and emotional strain associated with the task were impactful. Interestingly, while no overall sex differences emerged in the direction of mood change, males reported significantly lower baseline mood prior to the experiment. This baseline gap may reflect pre-existing differences in emotional state or expectations about task performance, though post-task mood converged across sexes.

Primary Appraisal Secondary Appraisal stress scores increased stepwise across phases, confirming that participants subjectively experienced greater stress as the task progressed from control to stress phase 1 and stress phase 2. The lack of sex differences in PASA responses suggests that, despite the observed differences in physiological and neural patterns (as seen in EEG and ECG results), subjective stress appraisal was comparable across males and females.

Performance measures mirrored these subjective effects. Error rates increased dramatically across phases, reflecting the growing cognitive demands and stress-related attentional disruption. Response times followed a non-linear trend: participants responded faster under moderate stress (stress phase 1), potentially due to heightened arousal, but slowed down under higher stress (stress phase 2), consistent with cognitive overload or disengagement. These patterns were similar across sexes, reinforcing that the task’s difficulty scaling and its impact on behavior were robust and comparable across groups.

Practice-related improvements were also modulated by stress context. During the control phase, error rates declined across task runs, indicating typical learning effects. This trend was blunted in stress phase 1 and reversed in stress phase 2, where early improvements gave way to performance degradation. These findings suggest that while participants can adapt and improve under low-stress conditions, stress impairs the application of learning especially under sustained high demand.

### 4.2 ECG features as reliable stress markers?

Raw HR data showed that females exhibited overall higher HR than males, consistent with sex-based differences in cardiovascular physiology—such as smaller stroke volume and greater resting HR.

In females, raw HR indicates that overall autonomic tone decreases with stress and then plateaus, suggesting a general suppression in cardiovascular activation once stress is introduced. The elevated HR observed during the control phase may also reflect anticipatory anxiety related to the upcoming math stressor. Such anticipatory responses are consistent with literature showing that mere anticipation of performance-related stress can activate the sympathetic nervous system (Roos et al., 2021). The progressive HR decline across stress phases could possibly also be linked to mental fatigue, as supported by findings from Matuz et al. (2021), who demonstrated that mental fatigue from prolonged cognitive engagement leads to reduced HR, reflecting diminished energetic arousal and decreased task engagement. Consistently, in our previous work (Riedl et al., 2023), we reported that videoconference fatigue—another cognitively demanding condition—was associated with decreased HR as well. While in Riedl et al. (2023) we observed significantly increased frontal and occipital T and A power associated with fatigue, herein we did not identify such changes with experimental phase (Figure 7), suggesting that the observed effects are likely not attributable to mental fatigue. We also did not find statistically significant differences in the BMIS scores for the adjectives “tired” and “active” before and after the experiment (for either males or females), which could have indicated the presence of fatigue. Furthermore, baseline-corrected data reveal that stress phase 1 still triggers a stronger phasic response relative to its own baseline – suggesting moderate stress briefly activates the system, while high stress leads to blunted reactivity. This supports the idea of an initial overactivation followed by physiological fatigue or reduced flexibility under continued stress exposure.

In males, raw HR declined from control to stress phase 1 and showed partial recovery in stress phase 2, suggesting some reactivation of autonomic activity under higher stress. However, baseline-corrected HR revealed a monotonic decrease in reactivity, with HR falling below baseline in both stress phases and reaching its lowest point in stress phase 2. This indicates that despite the apparent rebound in raw HR, the cardiovascular system remained less responsive relative to its pre-phase state. The combined pattern suggests reduced physiological flexibility or gradual disengagement in response to increasing cognitive demands.

Across all participants, a brief dip in HR was observed around the time of response onset–a characteristic cardiac deceleration typically attributed to parasympathetic activation. This transient bradycardia is commonly associated with focused cognitive engagement and motor preparation, serving to enhance attentional and sensorimotor control during response execution (Jennings et al., 1980; Jennings, 1982; Pfurtscheller et al., 2013; Skora et al., 2022; Henderson et al., 2024). After the brief parasympathetic deceleration, there is typically a reactivation of sympathetic nervous system activity as the individual executes the response or processes its outcome, which results in a post-response acceleration of HR. Skora et al. (2022) proposed a mechanistic explanation: event-related HR deceleration sharpens perceptual focus by reducing baroreceptor noise via vagal activation, facilitating more efficient sensorimotor control. After response execution, parasympathetic withdrawal and sympathetic rebound promote action and recovery.

Consistent with this view, multiple studies using choice reaction time tasks (Jennings, 1982; Zimmer et al., 1990) have shown anticipatory HR slowing that intensifies with task repetition, predicting faster but more impulsive responses. In error-related paradigms, (Danev and de Winter, 1971; Wimmer et al., 2024) found that correct performance was followed by HR rebound, while errors prolonged HR deceleration, suggesting prolonged parasympathetic engagement during error monitoring. Pfurtscheller et al. (2013) observed initial HR deceleration in both action and no-action conditions during motor imagery Go/No-Go tasks, with a quicker rebound in active trials, highlighting the role of movement preparation in cardiac dynamics. In tasks requiring increased cognitive load, Henderson et al. (2024) showed that higher difficulty led to earlier and more gradual HR deceleration, while Dennis and Mulcahy (1980) emphasized that HR deceleration reflects cognitive load during stimulus evaluation. Kaiser and Sandman (1975) found greater HR deceleration during the warning period of anagram problem-solving, with more difficult anagrams linked to higher HR deceleration, indicating greater attentional demands. Finally, De Pascalis et al. (1995) found that stress slowed HR deceleration before the probe stimulus in a visual recognition task, suggesting that stress reduces processing capacity, making it harder to prepare for stimuli.

In the present study, these prior findings are projected in the observed HR dip and rebound patterns, but with modulation by stress level and sex. Participants who felt more stressed had larger HR dips, showing that the body reacts more forcefully to higher perceived stress. Importantly, males and females managed these stress responses in different ways. Men tended to show earlier and longer-lasting HR dips, suggesting a strategy based on timing—they start reacting earlier and maintain a steady response over time. Women, on the other hand, showed larger dip and rebound magnitudes, especially during slower responses, indicating a strategy based on intensity—their bodies react more forcefully, though for a shorter duration.

These results underscore that temporal features of HR regulation may offer sensitive indices of physiological states that are not captured by average HR levels alone and further reveal sex-related differences in the timing and dynamics of autonomic responses (Table 3).

**TABLE 3:** Summary of ECG and EEG results.

Data	Aspect	Females	Males	All participants
ECG	Baseline HR (Raw)	Higher than males; possible anticipatory anxiety	Lower than females	___
	HR trend across phases	Decrease from control to stress, then plateau	Decrease in stress 1, partial rebound in stress 2 (raw)	Initial activation then reduced reactivity
	Baseline-corrected HR	Phasic response in stress 1; blunted in stress 2	Monotonic decrease below baseline across phases	___
	Response-locked HR dip	Larger dips and rebounds (intensity-driven)	Earlier, longer dips (timing-driven)	Brief dip followed by rebound post-response
	Interpretation	Suppression after stress onset; strong short-duration reactions	Steady, early engagement with reduced flexibility	Cardiac deceleration and rebound align with cognitive engagement
EEG	Frontal theta and beta power	Increased; linked to attentional control and cognitive load	Less prominent changes	Stress modulates T and B power in frontal regions
	Frontal T/B ratio	Sensitive marker across stress, performance, and PASA score	Not a consistent marker	___
	Gamma power	Moderate increase under stress	Strong increases across stress and errors; frontal/posterior	Stress increases G power, more so in males
	Delta power	Less affected	Decreased in central/parietal left hemisphere; reduced motor readiness	Stress may reduce MRCPs
	Alpha asymmetry	Left > right; linked to negative affect and emotional regulation	Left frontal dominance; right PFC activation under stress	Asymmetry modulated by stress in both sexes
	Performance link	EEG features correlate with performance and stress ratings	Less direct, more influenced by cumulative stress-performance effects	EEG markers reflect both stress and cognitive effort
	Mechanism interpretation	Engages localized, frequency-specific networks	Broader, high-frequency cortical activation patterns	Sex-dependent EEG strategies under stress

### 4.3 EEG features as reliable stress markers?

The EEG findings demonstrate that both task-induced and self-reported stress are associated with robust neural changes. Importantly, the overall patterns of stress-induced EEG power changes observed here closely mirror the t-map results reported in our previous work (Wriessnegger et al., 2024). This consistency across studies reinforces the robustness of these neurophysiological markers in capturing stress-related cognitive and affective dynamics. The nature, however, and distribution of these changes differ between females and males. While similar topographic patterns were observed across sexes, the underlying spectral and lateralization profiles point to distinct neurophysiological strategies. Specifically, D power significantly decreased in men over central and parietal electrodes especially on the left hemisphere. Studies show greater hemispheric lateralization in males, while females exhibit more bilateral activation, especially in prefrontal and limbic areas. This may correspond to more synchronized theta or alpha activity in females during emotionally charged tasks and increased gamma or beta in males due to lateralized, goal-oriented processing (Hodgetts and Hausmann, 2022).

In females, stress consistently modulated frontal T, B power, and the T/B ratio–features tightly linked to cognitive control and attentional regulation. This aligns with previous findings showing that frontal-midline theta and beta oscillations are highly sensitive to cognitive load and stress (Putman et al., 2014). Wen and Aris (2020) and Wen et al. (2020) also reported a negative correlation between T/B and stress. Herein, the frontal T/B ratio emerged as a sensitive marker across experimental phase, PASA stress scores, and error rate, which may reflect increased attentional demands and emotional processing.

Prior studies already reported on sex-related neurotransmitter system differences, specifically serotonin, dopamine, and GABAergic systems are modulated differently by sex hormones, impacting stress reactivity and EEG markers. For example, estrogen upregulates serotonin and GABA receptors, which may contribute to increased alpha/theta power, associated with emotion regulation and internal attention (Bangasser and Valentino, 2014; Bangasser and Wicks, 2017). Neurochemically, estrogen modulates limbic-frontal interactions, enhancing women’s sensitivity to emotional and cognitive stressors and likely contributing to the observed EEG profiles. Neurohormonal influences, particularly the modulatory role of estrogen and testosterone, likely shape oscillatory dynamics by influencing cortical excitability and connectivity in regions such as the PFC and limbic system (Shansky et al., 2004; Barth et al., 2015). Estrogen has a profound effect on brain function, particularly in modulating limbic system activity (amygdala, hippocampus) and PFC regions responsible for emotional regulation and executive control. Whereas estrogen enhances synaptic plasticity, cortical excitability, and can influence oscillatory patterns, especially in theta and alpha bands, testosterone is associated with greater activation in brain areas linked to goal-directed behavior and risk-taking, potentially increasing beta and gamma activity under stress.

Structurally, females show more bilateral activation during cognitive-emotional tasks, which may relate to greater theta and alpha power, supporting emotion-focused coping. Males, on the other hand, showed a different pattern of modulation. Gamma power was the most consistently affected feature across all predictors, with particularly strong increases under stress and high error rates. This aligns with prior findings showing that prefrontal G power significantly increases under acute psychosocial stress (Minguillon et al., 2016). Similarly, recent work by Pei et al. (2023) demonstrated that task difficulty systematically increases G band amplitude, particularly in posterior regions, reflecting elevated cortical arousal and high-load cognitive processing. Furthermore, males often exhibit lateralized activity patterns associated with heightened beta and gamma power, supporting rapid, action-oriented coping mechanisms (Bangasser and Valentino, 2014). This also aligns with evolutionary theories proposing the “fight-or-flight” stress responses wherein sex-specific EEG activation may represent adaptive neural strategies to environmental stressors (Taylor et al., 2002; Gur and Gur, 2016). Males may prioritize rapid problem-solving and response execution, reflected in more high-frequency EEG activity, like the observed gamma power. Although G activity increases can sometimes reflect residual EMG contamination, we consider this unlikely to fully account for our findings. First, our ICA and artifact rejection steps specifically targeted muscular sources. Second, the topographical patterns we observe, which are characterized by more global increases, do not match the typical peripheral distributions of muscle signals near the scalp, and these global increases were more pronounced in male participants than in female.

We also observed significant decreases in D power in central and parietal regions under stress, particularly in the left hemisphere. This lateralized D reduction (i.e., mainly in the left hemisphere) may reflect decreased amplitudes of movement-related cortical potentials (MRCPs), which reside in the D band and are associated with motor preparation processes involved in generating a response (Shibasaki et al., 1980; Shibasaki and Hallett, 2006). These findings suggest that stress may impair or reduce the efficiency of motor preparation processes in men. Mental stress and anxiety have been shown to attenuate MRCPs (Glanzmann and Froehlich, 1984), particularly the Bereitschaftspotential, which reflects motor preparation (Shibasaki et al., 1980). High-anxiety individuals exhibit significantly reduced Bereitschaftspotential amplitude and diminished prefrontal negativity, indicating impaired preparatory brain activity. This reduction is linked to weakened top-down attentional control and slower, less accurate responses during tasks (Olsen et al., 2021). However, studies also show that task-related feedback can partially restore MRCP amplitude and normalize performance in anxious individuals, suggesting that stress-induced suppression of motor preparatory signals may be modulated by cognitive context and external cues (Mussini and Di Russo, 2023). Overall, these findings highlight that anxiety impairs the neural readiness for movement, which can be detected through reduced MRCP amplitude.

In females, experimental phase, stress and error rate induced higher A power on the left hemisphere (i.e., greater right hemispheric activation) especially in frontocentral and centroparietal sites. Greater right hemispheric activation is linked to withdrawal and negative affect, while frontocentral asymmetries are consistent with greater integration of emotional regulation and task-related cognitive effort (Smith et al., 2017). In contrast to females, A asymmetry effects in males were present for stress-related predictors but not for performance, and were more fronto-occipitally distributed, with A power being more pronounced in the left frontal areas and reduced in the left occipital areas. This is supported by the work of Earle and Pikus (1982) who found that in males A asymmetry was not significantly correlated with task performance, unlike in females. Furthermore, our findings align with Wang et al. (2007), who showed that stress in males primarily activated the right PFC and suppressed the left orbitofrontal cortex, reflecting an executive-focused “fight-or-flight” neural response, whereas females exhibited stronger limbic system activation (including the insula, striatum, and anterior cingulate cortex) linked to emotional and integrative stress processing. Although no significant asymmetry effects were found for error rate alone in males, the similarity between the error rate and experimental phase topographies suggests that performance variability may have contributed to experimental phase effects when combined with stress-related factors. These results indicate that, in males, EEG asymmetry shifts are likely driven by the cumulative interaction of subjective stress and task performance, rather than performance decline alone.

While both sexes show EEG sensitivity to stress and performance, the underlying mechanisms seem to differ (Table 3). Females appear to engage more frequency-specific and functionally localized control systems, while males rely on broader, high-frequency activation patterns. Alpha asymmetry offers a common axis of modulation, but with different behavioral correlates and topographies across sexes. These distinctions have practical implications for the design of neuroadaptive interfaces and stress monitoring systems, which may require sex-specific models or feature weighting to accurately capture neural signatures of mental effort and performance.

### 4.4 Limitations and future work

It is worth noting that HRV is often considered a key physiological marker in stress-related research (Hickey et al., 2021). However, in the present study, our analysis focused solely on HR, as several methodological limitations prevented reliable HRV estimation. Specifically, the analysis window of 2.5 s per trial was too brief to derive meaningful HRV measures. Although we attempted to compute HRV indices using the full duration of each experimental phase, variability in phase lengths, driven by individual differences in response times, limited our analysis to a minimum segment of approximately 3 min, starting from the onset of each phase. According to Shaffer and Ginsberg (2017), even short-term HRV assessments typically require at least 5 min of continuous data. Our HRV analysis (specifically using low-frequency/high-frequency HR power, and root mean square of successive differences metrics) yielded no significant findings, likely due to the insufficient duration of usable data segments.

Another methodological limitation concerns the duration of the baseline recordings. While we applied baseline correction using 10-s resting segments before each phase, longer baseline periods (e.g., 2–5 min) could provide more stable reference. Our design prioritized phase-specific normalization to account for immediate physiological state shifts. Nevertheless, longer resting-state baselines, especially at the beginning of the experiment, would strengthen absolute comparisons and improve between-subject normalization.

While our findings suggest potential sex-related differences, it is important to acknowledge that the sample size limits the generalizability of these results. As such, the observed effects should be interpreted with caution and considered exploratory. Furthermore, we did not record menstrual cycle phase, contraceptive use, or hormone levels in our female participants, which are known to affect EEG and autonomic stress responses (Solís-Ortiz et al., 1994; Baehr et al., 2004; Bazanova et al., 2014; Solís-Ortiz et al., 2019; Torbaghan et al., 2023; Gaižauskaitė et al., 2024; Hemakom et al., 2024), the lack of hormonal-status control may limit the specificity of our sex-based comparisons. Future studies should include these measures to clarify hormonally driven effects.

The current study also relies solely on mathematical stress tasks, which may not fully capture real-world stress responses. Future paradigms should consider social, emotional, or multitasking stressors. Finally, although the study was motivated by interest in neurocardiac correlates of stress, EEG and ECG features were analyzed independently. A promising direction for future research involves implementing joint EEG–ECG modeling (using e.g. multivariate autoregressive models (Kostoglou et al., 2019)) to better capture the integrated dynamics of brain–heart interactions under stress.

Additionally, our ECG preprocessing and QRS detection relied on the classical Pan–Tompkins algorithm (Pan and Tompkins, 1985; Sedghamiz, 2025). Although visual inspection confirmed that detected QRS complexes align accurately with true R-peak locations in our dataset, the algorithm’s fixed filter parameters, hard-coded decision thresholds, and limited adaptability render it less robust than more recent detection methods (Martínez et al., 2004; Zhang and Lian, 2009; Abibullaev and Seo, 2011). We therefore acknowledge these constraints as limitations of the present study and plan to address them in future work.

One further limitation is the missing formal statistical testing of sex × predictor interactions which would strengthen the interpretation of sex-specific EEG findings. However, given the high dimensionality of the EEG data, across many channels and frequency bands, introducing interaction terms would substantially increase model complexity and make the results difficult to visualize and interpret. For this reason, we opted to present EEG topographies separately for females and males to highlight qualitative differences, while keeping the analysis tractable and interpretable. We plan to explore formal cross-sex statistical models in future work.

## 5 Conclusion

Mental stress is typically assessed by doctors or experts through interviews and psychological questionnaires. However, these methods rely on subjective responses, which can lead to inaccurate assessments and hinder effective treatment. Moreover, such evaluations are often conducted long after the stress exposure, by which time mental or physical damage may have already occurred. In contrast, assessing stress through physiological signals, directly linked to autonomic nervous system responses, offers a more immediate and objective approach. Summarizing our findings, the questionnaire and performance data validated the stress manipulation and revealed distinct behavioral consequences of increased cognitive-emotional load. While males and females reported similar subjective experiences and showed no behavioral performance differences, earlier baseline mood differences and distinct neurophysiological patterns suggest that the same external stressors may be internally processed through different pathways. Females seem to use a combined emotional and cognitive control strategy, involving frontal brain networks and stronger HR reactions, while males rely more on executive and vigilance control, shown by frontal and occipital brain activity and earlier HR adjustments. This underscores the value of integrating physiological, subjective, and behavioral data to fully capture individual and sex-specific responses to cognitive stress.

## Data Availability

The raw data supporting the conclusions of this article will be made available by the authors, without undue reservation.
